# Application of Universal Stress Proteins in Probing the Dynamics of Potent Degraders in Complex Terephthalate Metagenome

**DOI:** 10.1155/2013/196409

**Published:** 2013-09-10

**Authors:** Andreas N. Mbah, Raphael D. Isokpehi

**Affiliations:** Center for Bioinformatics & Computational Biology, Department of Biology, Jackson State University, Jackson, MS 39217, USA

## Abstract

The culture-independent strategies to study microbial diversity and function have led to a revolution in environmental genomics, enabling fundamental questions about the distribution of microbes and their influence on bioremediation to be addressed. In this research we used the expression of universal stress proteins as a probe to determine the changes in degrading microbial population from a highly toxic terephthalate wastewater to a less toxic activated sludge bioreactor. The impact of relative toxicities was significantly elaborated at the levels of genus and species. The results indicated that 23 similar prokaryotic phyla were represented in both metagenomes irrespective of their relative abundance. Furthermore, the following bacteria taxa Micromonosporaceae, *Streptomyces, Cyanothece* sp. PCC 7822, *Alicyclobacillus acidocaldarius, Bacillus halodurans, Leuconostoc mesenteroides, Lactococcus garvieae*, Brucellaceae, *Ralstonia solanacearum, Verminephrobacter eiseniae*, *Azoarcus, Acidithiobacillus ferrooxidans, Francisella tularensis, Methanothermus fervidus,* and *Methanocorpusculum labreanum* were represented only in the activated sludge bioreactor. These highly dynamic microbes could serve as taxonomic biomarkers for toxic thresholds related to terephthalate and its derivatives. This paper, highlights the application of universal stress proteins in metagenomics analysis. Dynamics of microbial consortium of this nature can have future in biotechnological applications in bioremediation of toxic chemicals and radionuclides.

## 1. Introduction

Terephthalate (TA) is also known chemically as polyethylene terephthalate (PET) or terephthalic acid (PTA) (1,4-benzenedicarboxylic acid) [1]. Terephthalate and its isomers are among top 50 chemicals manufactured in the world [2, 3]. The chemical derivatives are used in the manufacturing of textile, polyester films, adhesives, coatings, and polyethylene terephthalate bottle [4]. The terephthalate wastewater is typically treated by aerobic biological systems [5, 6]. The anaerobic treatment requires less energy and nutrients than traditional aerobic processes and has become an attractive alternative [7]. Using anaerobic bioreactors, organic compounds are transformed into methane and carbon dioxide through a complicated network of a consortium of different types of bacteria. There are documented studies on these metabolic networks based on anaerobic degradation of some known agroindustrial wastewater constituents such as alcohols and fatty acids [8, 9]. 

The anaerobic sludge bioreactor has been shown to be very successful in treating terephthalate containing wastewater [10, 11]. The microecosystem is composed of acetogenic bacteria which are good degraders of complex organic compounds, converting them to intermediate mixture of formate, hydrogen, and acetate. The methanogenic archaea present will then mineralize the intermediates to methane and carbon dioxide [12–16]. Due to favorable energy in the fermentation step from terephthalate to acetate, the microbial population interacts syntrophically and requires the methanogenesis step as a coupling reaction to drive the process [17]. It has also been suggested that within the methanogenic consortium, terephthalate is degraded via decarboxylation to an intermediate benzoyl-CoA and later to acetate and hydrogen which are mineralized to methane and carbon dioxide [18, 19]. The degradation of terephthalate to acetate and hydrogen was due to the fermentative population, while the methanogenesis process was the sole responsibility of both hydrogenotrophic and acetoclastic methanogens. However, this pathway remains unconfirmed due to the difficulty in isolating synthrophic bacteria [8, 18]. 

Many petrochemical industries are producing a wide range of chemical intermediates, plastics, and synthetic rubber from petroleum raw materials, thus generating enormous amount of toxic cyanide-containing wastewater [20], and many other varieties of toxic compounds that are considered potential stressors to the anaerobic degrading microorganisms [21–23]. As such the microbial degrading consortia of terephthalate are under enormous amount of environmental stress. A phenotypic feature crucial to an organism's survival is the ability to response and adapt to unfavorable or stressful environment. A protein family known to enable bacteria, archaea, fungi, and plants to respond to unfavorable or stressful environment is the universal stress protein (USP) family [24, 25]. The universal stress proteins (USPs) are found in diverse group of organisms like archaea, eubacteria, yeast, fungi, and plants (Pfam accession number PF00582), and encompass a conserved group of proteins whose expressions are triggered by a large variety of environmental insults [26]. This variety of stress conditions may include starvation of one of the following nutrients: carbon, nitrogen, phosphate, sulfate, and the required amino acid and the presence of a variety of toxicants and other agents including heavy metals, oxidants, acids, antibiotics, heat shock, DNA damage, and uncouplers of the electron transport chain [27, 28]. 

The transcripts from universal stress proteins are considered low abundant transcripts [24, 29] and are induced to perform specialized functions triggered by environmental stressors as indicated above. This implies that USPs are not housekeeping genes. Multiple USP genes may be encoded in a genome and are expressed to circumvent varying insults [26]. Species-specific USP genes may differ completely in both their subcellular locations and functions. This specificity indicates that the expressed USP genes depend on the triggers in question and the functions to be performed in the immediate environment. In an extreme and noxious environment such as terephthalate wastewater and bioreactor systems, the USPs genes could be acting as both extracellular sensor to the environmental insult (terephthalate) [30, 31] and as a cellular protector to the organism's degrading enzymes from possible toxicant denaturing effect [32].

The USP genes and their signal peptides are very sensitive to the stressor(s) threshold value(s), and thus these genes will be expressed at a minimum threshold value, and above the maximum threshold value the organism will be extinct from the system. Only organisms with highly equipped USP apparatus will survive higher threshold values. This implies that the threshold value of an environmental insult can be a dynamic parameter for the microbial consortium, with the appearance or disappearance of particular organism(s) serving as biomarker(s) of stressor levels. The wastewater is expected to have a higher threshold of terephathalate toxicants and only organisms with well-adapted USP genes will proliferate at this toxic threshold. As the wastewater is treated in the activated sludge, its toxicity decreases continuously until the accepted treatment level is attained. Therefore the dynamic of the microbial consortium in the bioreactor can be correlated or calibrated to the stressor (terephthlate toxicant) value in the biotreatment system.

A metagenome analysis has been proven as an effective method for retrieving interesting microbial populations from complex ecosystems [33, 34]. This paper reports research investigation to elucidate the dynamics of potent degrading microbial populations in a bioreactor treatment plant containing terephthlate toxicant. We have used bioinformatics strategies on metagenomics data to identify microbes expressing the universal stress protein's genes from the terephthalate degrading microbial community. We used GOLD ID: Gm00012 metagenome library constructed from both wastewater and terepthalate bioreactors samples. The results reveal that the dynamics of the microbial consortium in both highly toxic wastewater and the less toxic activated sludge could be potential biomarkers of toxicity thresholds. To our knowledge, this paper is the first report on applying universal stress protein's genes as initial filter for comparing metagenomes. The findings could have biotechnology applications in the bioremediation of toxic chemicals and radionuclides.

## 2. Methods

**Figure 1:**
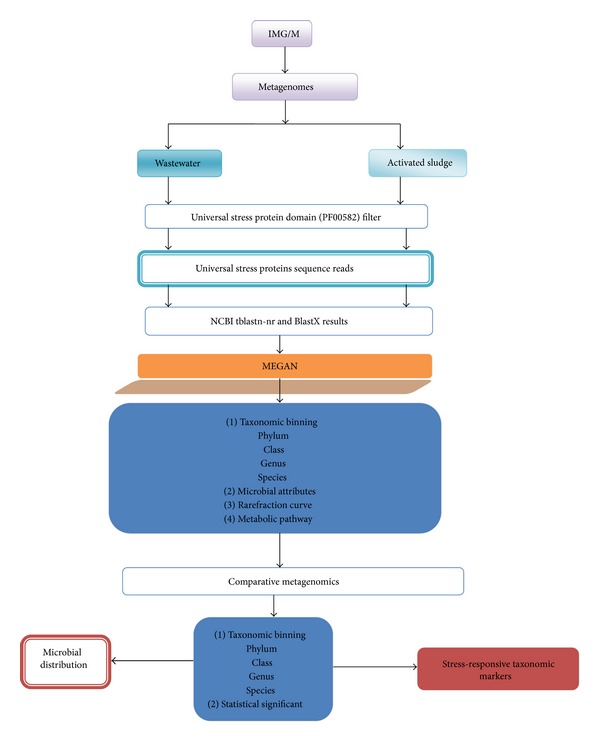
Overview of the procedure identifying microbial taxonomic distribution and universal stress protein biomarkers.

The procedure for identifying microbial taxonomic distribution and universal stress protein biomarkers are summarized in Figure 1. Each step is described in the sentences.

### 2.1. Read Extraction and Sequence Similarity Search

The universal stress protein reads were extracted using Pfam annotation. The finished metagenomes were retrieved from the Integrated Microbial Genomes with Microbiome Samples (http://img.jgi.doe.gov/cgi-bin/m/main.cgi) using the Pfam domain accession Pfam00582. We obtained universal stress protein sequence reads from the terephthalate activated sludge and terephthalate wastewater metagenomes. The Pfam00582 gene function was used as filter for reads corresponding to the universal stress protein expressing microbial community. To determine the taxonomic distribution of the source organisms for the universal stress proteins and their functional annotations in each metagenome sample, we ran tblastn comparison against the National Center for Biotechnology Information (NCBI) nonredundant nucleotide database (ncbiN-nr) and blastx against the NCBI nonredundant Protein Database (ncbiP-nr) at default parameters [35] and followed the steps in the workflow chart (Figure 1).

### 2.2. Metagenomic Analysis and Visualization

#### 2.2.1. Taxonomic Binning

MEGAN (“MEtaGenomeANalyzer”) software version 4.70.4 was used to process the NCBI BLAST output file for taxonomic sources of the sequences. The processing was based on the NCBI taxonomy embedded in MEGAN with default lowest common ancestor (LCA)-parameters (min. score: 35, top percent: 10.0 and min. support: 5, and disable taxa: 9). The LCA algorithmin MEGAN randomly chooses 10%, 20%,…, 100% of the total number of reads as subsets. For each of these random subsets the number of leaves (hit with at least 5 reads (min support) is determined. The tblastn and blastx search results were loaded into MEGAN and applied the LCA algorithm to compute the assignment of reads to taxa and estimate the USP expressing taxonomical content of each metagenome. We mapped the taxon information of significant matches at phylum, class, genus, and species levels for the two metagenomes. We used all ranks of the NCBI taxonomy, placing more conserved sequences higher up in the taxonomy (i.e., closer to the root) and more distinct sequence onto nodes that are more specific (i.e., closer to the leaves, which represent species and strains).

### 2.3. Comparative Visualization and Statistical Significance of Reads

The different datasets were brought together and compared for taxonomical and functional attributes under a multiple comparison tree. The metagenomes were compared at the levels of phylum, class, genus, and species including their functional attributes using normalized read count. The comparison was done by generating different types of interactive visualization using bar and pie charts where each node in the NCBI taxonomy is shown as a pie or bar chart indicating the number of normalized reads from each dataset that have been assigned to that node. In this work we used the Holm-Bonferroni correction [36, 37] significance test at 1% confidence interval for both the up test and the down test. The *P* value was automatically generated by MEGAN. The up test is visible as a black bar to the left of nodes for phylum and specie comparisons. The down test on the other hand incorporates Pearson's *χ* ^2^-test to compare the distribution of the two datasets on the children of a particular node. If the *P*-value of the up test is below the critical level (0.01), then the part of the node that faces the parent will be highlighted, whereas a significant *P*-value for the down test will result in the part of the node that faces the children to be highlighted. 

#### 2.3.1. Rarefraction Curve

An important question in metagenomic sampling is whether the level of sequencing performed for a given sample is sufficient to capture the most abundant taxa. We addressed this by plotting the discovery rate of the metagenomes, also called rarefraction analysis or species richness. The species richness was estimated by rarefaction analysis in MEGAN as indicated by [38]. The MEGAN program uses an LCA-algorithm to bin reads to taxa based on their blast-hits. This results in a rooted tree where each node represents a taxon as stated above. The leaves in this tree are then used as operational taxonomic units (OTUs) in the rarefaction curve analysis. The program randomly chooses 10%, 20%,…, 100% of the total number of reads as subsets. For each of these random subsets the number of leaves (hit with at least 5 reads (min. support) is determined. This random subsampling is repeated 20 times, and then the average value is used for each percentage. Our analysis was evaluated at the most resolved level of the NCBI taxonomy to capture as much of the richness as possible. At this level, the leaves were mostly strains and species. We analyzed all taxa in the metagenomes (Bacteria, Archaea, Eukaryota, viruses, and environmental sequences). The graph obtained can be used to give a rough estimate on how many additional species are likely to be discovered if the number of reads are increased by a certain factor.

#### 2.3.2. Metabolic Pathway Analysis

The MEGAN program automatically calculates functional classification of the reads using either the SEED comparative genomics environment or the Kyoto Encyclopedia of Genes and Genomes (KEGG) classification or both. The results can be interactively viewed and inspected. In SEED classification MEGAN attempts to map each read to a SEED functional role, using the highest scoring BLAST match to a protein sequence for which the functional role is known. The SEED classification will be depicted as a rooted tree whose internal nodes represent the different subsystems and whose leaves represent the functional roles. For KEGG analysis the program attempts to match each read to a KEGG orthology (KO) accession number using the best hit to a reference sequence for which a KO accession number is known. This information is then used to assign reads to enzymes and pathways. The KEGG classification will be represented by a rooted tree whose leaves represent different pathways. Each pathway can also be inspected visually, to see which reads were assigned to which enzymes. Both KEGG and SEED classifications were annotated in the metagenomes analyzed.

## 3. Results and Discussion

### 3.1. The Universal Stress Protein Reads and Sequence Similarity Searches

The PF00582 gene filtered was used to extract genes encoding universal stress proteins from both metagenomes. The taxonomical classification of the reads queried against the NCBI nonredundant Nucleotide Database (ncbiN-nr) yielded file size of 19.41 GB containing 6,735 reads from wastewater and 13.6 GB containing 4,574 reads from the activated sludge. These reads were used for taxonomic binning. Furthermore, the BlastX query against the NCBI nonredundant Protein Database (ncbiP-nr) resulted to 7,133 reads for the wastewater and 5,475 reads from the activated sludge samples. In 1992, Nyström and Neidhardt [27] reported the cloning, mapping, and nucleotide sequencing of a monocistronic gene in *Escherichia coli* encoding a small (13.5 kDa) cytoplasmic protein with increased synthesis during growth inhibition or presence of toxic agents. The gene was designated UspA, and subsequent mutant-based analysis of the gene led to the proposal that the encoded protein may have a general protective function related to the growth arrest state [39]. Genomic data have been used to identify genes encoding the USP domain in the Archaea, Bacteria and Eukaryotes [26, 40–42]. This report represents a useful application of data mining and integration to generally underutilized USP genes expression in metagenomics studies and their possible application in bioremediation biotechnology. The common effect of toxic stress to an organism is to denature its proteins. Stress proteins are stress-inducible and they respond to a variety of environmental stressors through activation of various intracellular signaling pathways. All known stresses, if sufficiently intense, induce expression of these proteins. A common aspect of these inducing stresses is that they result in proteins having nonnative conformations [32, 43]. 

### 3.2. Metagenomic Analysis and Visualization

**Figure 2:**
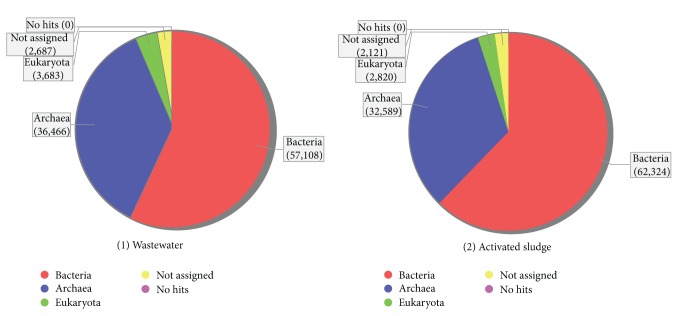
The distribution reads assigned to the domains Bacteria and Archaea. The entire reads show that there are significant differences in the proportion of reads assigned to the domains Bacteria and Archaea from the two metagenomes.

The BLAST output files were analyzed according to NCBI taxonomy in MEGAN using default LCA-parameters (min. score: 35, top percent: 10.0 and min. support: 5, and disable Taxa: 9). The distribution of the entire sequence (reads) shows that there are significant differences in the proportion of reads assigned to the domains Bacteria and Archaea for the two metagenomes (Figure 2). In the wastewater metagenome 57,108 reads were assigned to Bacteria and 36,466 reads to Archaea, while in the activated sludge metagenome 63,334 reads were assigned to Bacteria and 32,589 reads to Archaea. The presence of the prokaryotic community structure in both metagenomes suggests that terephpthlate degradation follows a two-step process through the syntrophic association between fermentative bacterial groups, which convert terepthalate to acetate and hydrogen and the methanogens, which convert acetate and hydrogen to final gaseous products (CH_4_ and CO_2_) [12–16]. An important feature of MEGAN is the ability to interactively collapse or expand a tree at different levels of the taxonomy, making it possible to start at a high-level view and then drill down to a low-level comparison.

In this research we have used the Holm-Bonferroni correction [36, 37] significance test at 1% confidence interval for both the up test and the down test. This version corrects results that could have been identified by chance and thereby represent uninformative data, thus giving a strong indication of significant difference between the metagenomes. Holm-Bonferroni correction analysis of the binned reads showed a striking difference between the two metagenomes on the abundance of the phyla and species reads. The MEGAN software uses two directed homogeneity tests called the up test and the down test to get an impression of how significant the two datasets differ as described in [44]. The test provides answers to two questions. (i) Is there a significant difference in the proportions of occurrences on a particular node in the two datasets? (ii) Is there a significant difference in the distribution of reads among the children of a particular node in the two datasets? The up test will answer the first question whether the proportion of assignments at a node as a fraction of its parent is significantly different between the two datasets. It uses the two-sample *t*-test, with the null hypothesis being that the two fractions are equal. The *P*-value thus describes the probability that the two fractions are from the same distribution, that is, that the corresponding organisms are equally abundant in both environments. The thickness of the black highlighting is logarithmically proportional to the significance.

**Figure 3:**
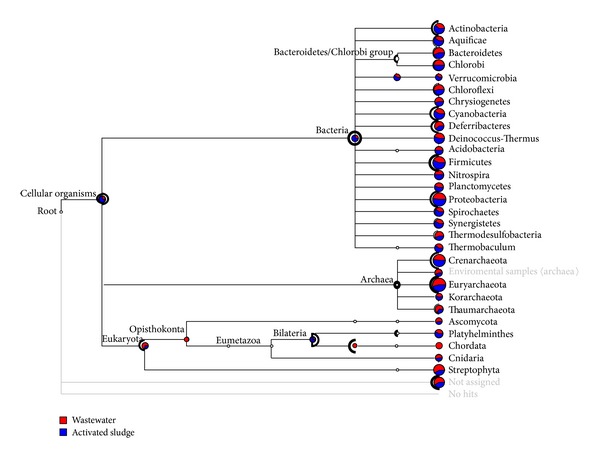
The distribution of reads assigned at phylum level. Holm-Bonferroni correction was used for significance test at 1% confidence. The thickness of the black highlighting is logarithmically proportional to the significance. A total of 23 similar prokaryotic phyla were represented in both metagenomes irrespective of their relative abundance. In the bacteria, the Proteobacteria was the most enriched group followed by Firmicutes and Bacteroidetes/Chlorobi group in that order. In the Archaea, the Euryarchaeota was also significantly followed by Crenarchaeota.

A total of 23 similar prokaryotic phyla were represented in both metagenomes, but their individual assigned reads showed remarkable significant differences (Figure 3). In the bacteria, the Proteobacteria was the most enriched group [45, 46] followed by Firmicutes and Bacteroidetes/Chlorobi group in that order. In the Archaea, the Euryarchaeota was significantly abundant [47–49] followed by Crenarchaeota. These findings are in accordance with previous characterization of the microbial consortia in laboratory-scale terephthalate systems. For example, most clones were affiliated to the delta class of the Proteobacteria which were closely affiliated with bacterial genera *Syntrophus* and *Smithella* that form syntrophic relationships with methanogens to degrade aromatic compounds such as benzoate [46]. Another research investigation observed that the archaea found in the syntrophic granule were all close relatives of methanogens in the Euryarchaeota and were closely affiliated to the genera *Methanosaeta, Methanospirillum* and *Methanogenium*, and the order *Methanomicrobiales* [45]. Concurrently other researchers reported that both acetoclastic *Methanosaeta* spp. and hydrogenotrophic methanogens (*Methanospirillum*, *Methanobacterium* and *Methanobrevibacter*) are frequently found in anaerobic sludge granules [47, 48] and are suggested to be important for sludge granulation [49]. This finding will further support the two-step degradation process of terephpthlate under mesophilic methanogenic conditions [12–16] with delta Proteobacteria and syntrophic methanogenic Euryarchaeota the major fermentative bacterial populations.

**Figure 4:**
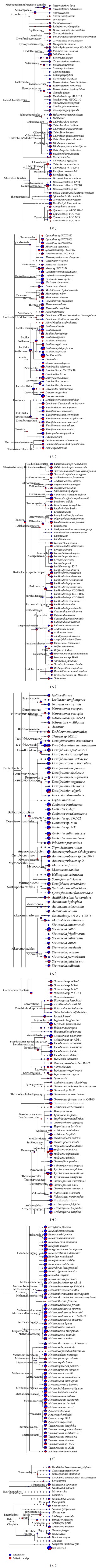
Dynamic of specie population from both metagenomes. The wastewater community is blue, and activated sludge population is red. The thickness of the black highlighting is logarithmically proportional to the significance. The following bacteria taxa were present only in the activated sludge Micromonosporaceae, Streptomyces, Cyanothece sp. PCC 7822, *Alicyclobacillus acidocaldarius*, *Bacillus halodurans*, *Leuconostoc mesenteroides*, *Lactococcus garvieae*, Brucellaceae, *Ralstonia solanacearum*, *Verminephrobacter eiseniae*, Azoarcus, *Acidithiobacillus ferrooxidans*, and *Francisella tularensis*. The Archaea species *Methanothermus fervidus* and *Methanocorpusculum labreanum* were not also represented in the highly toxic wastewater.

The impact of the relative toxicity was significantly elaborated at the levels of both the genus and species (Figures 3 and 4). We are interested in the dynamic of microbial population and their future applications in environmental bioremediation, as such we are focusing on the lower toxic activated sludge metagenome. Terephthalate and derivatives compounds such as benzoate, methyl benzoate, and other intermediate toxic chemicals have profound impact on the microbial community leading to significantly population diversity. In the lower toxic activated sludge community, the microbial genera that appeared in the activated sludge which were not represented in the highly toxic wastewater were Micromonosporaceae, *Streptomyces*, *Leuconostoc*, Brucellaceae, *Verminephrobacter*, and *Azoarcus*. In the Archaea genera, *Methanothermus* and *Methanocorpusculum* were not represented in the highly toxic wastewater. The following species of bacterial were present only in the activated sludge Micromonosporaceae, *Streptomyces*, *Cyanothece* sp. PCC 7822, *Alicyclobacillus acidocaldarius, Bacillus halodurans, Leuconostoc mesenteroides, Lactococcus garvieae*, Brucellaceae, *Ralstonia solanacearum*, *Verminephrobacter eiseniae*, Azoarcus, *Acidithiobacillus ferrooxidans,* and *Francisella tularensis*. The Archaea species* Methanothermus fervidus* and *Methanocorpusculum labreanum* were not also represented in the highly toxic wastewater (Figure 4). These highly dynamic microbes could represent the stress responsive taxonomic biomarkers for terepthalate threshold levels.

One of the interesting features of both metagenomes was the selective dynamics noticed at the family, genus and species levels. A key environmental factor influencing the dynamics of the microbial consortia and the eventual expression of the universal stress protein is the toxic stressor [27, 39]. We suggest that the decrease in the concentrations of terephthalate and its derivative after syntrophic methanogenic degradation [15, 46] accounts for the emergence of activated sludge specific species. The shift in the entire prokaryotic abundant in wastewater compared to activated sludge might be due to continuous depletion of the specific toxic compounds [50]. 

**Figure 5:**
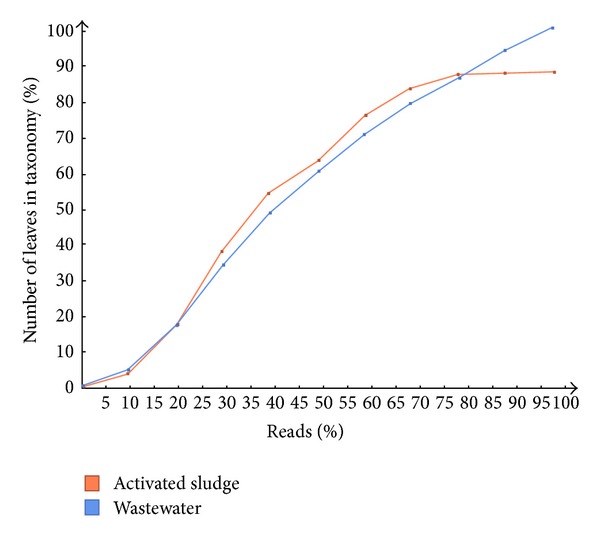
Rarefaction curve: the plots show the rarefaction curves of annotated species richness. It indicates the total assigned taxa (leaves) in percentage detected in the wastewater and activated sludge.

Rarefaction analysis was performed at the most resolved species level of the NCBI taxonomy in MEGAN. This illustrates the taxonomic richness detected in both metagenome samples (Figure 5). The plot shows the rarefaction curves of annotated species richness and the total assigned taxa (leaves) in percentage detected in both wastewater and activated sludge metagenomes. 

Both metagenomes curves rise very quickly at first, but the activated sludge then levels off as more new species are found per unit percentage of sequences sampled. The steep slope rarefaction curves for wastewater community indicated that not all the taxonomic richness had been accounted for and that a large fraction of the species diversity remains to be discovered and more intensive sampling is likely to yield additional species. Conversely, the activate sludge flattened out and indicates that complete taxonomic richness had been attained. From rarefaction analysis the high number of sequence reads for wastewater could be one of the contributing factors to the imbalance of the number of microbial families and species between both metagenomes. Another potential factor could be the shift in concentration gradient of terephthalate and its associated toxicants as more of these toxic chemicals become degraded. With time this could be pivotal to the emergence of the activated sludge unique families and species. Previous research has shown that the chemicals present in terephthalate wastewaters (i.e., terephthalic, phthalic, benzoic, trimellitic, and acetic acids), with the exception of p-toluic acid, are readily degradable with time in the bioreactor [50]. This process will significantly reduce the toxic concentrations and hence enriches the families and species that are specific to the activated sludge microbial consortium. However, the oxidation of terephthalate to acetate and hydrogen is an endergonic reaction unless coupled to methanogenesis reactions that it further convert those intermediates to the final gaseous products [13]. The shift in the entire prokaryotic abundance between wastewater and activated sludge might be accounted by the continuous depletion of toxic products [50].

#### 3.2.1. Metabolic Pathway Analysis

**Figure 6:**
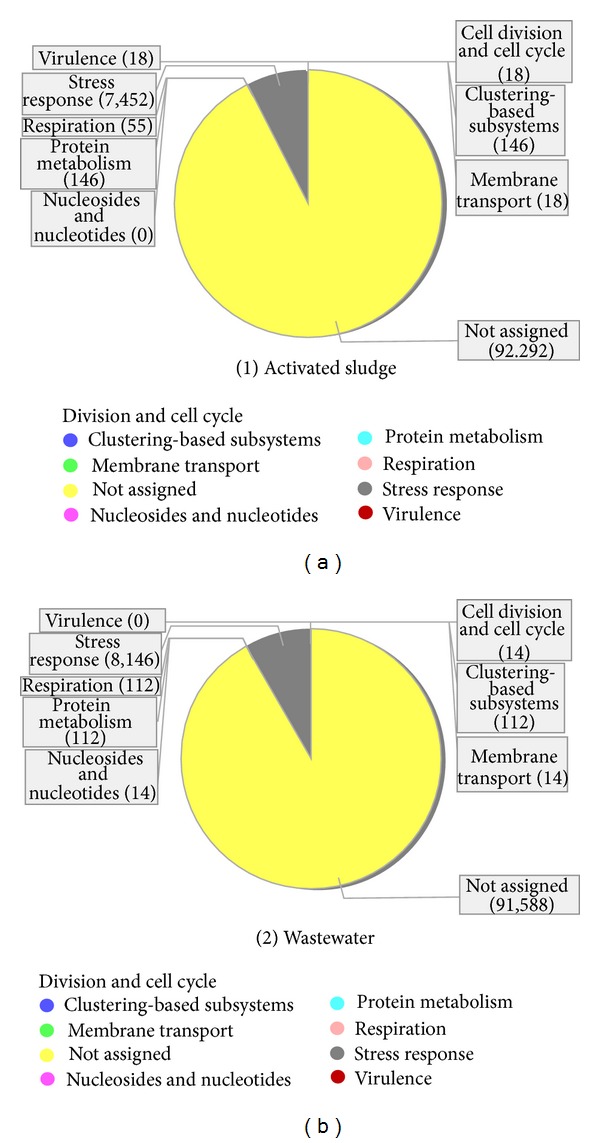
The comparative visualization showing the mapping of eight SEED terms.

To gain insight into the pathways, we annotated the reads from each metagenome to the KEGG and SEED terms mapped in MEGAN. The comparative visualization shows the mapping of eight SEED terms (Figure 6). The virulence term (TypeV pilus) was annotated to the activated sludge metagenome irrespective of the high reads abundance of pathogenic prokaryotes assigned to the wastewater sample. This indicates that the pathogenic microbes in the wastewater do not express the TypeV pilus virulence gene. In the terephthalate activated sludge bioreactor, incorporation of porous plastic biomass support particles (BSP) has resulted in the increased percentage of chemical oxygen demand (COD) reduction which was found to be related to the increase in biomass formation [20] and thus encourages the formation of prokaryotic biofilm during perturbation such as inadequate operational conditions to enrich the microbial consortia [51].

In *Escherichia coli* a global regulator had been identified which controls genes related to stress response, biofilm formation, and virulence by recognizing curved DNA and by silencing acquired genes [52]. Biofilm formation plays a critical role in the pathogenesis and is correlated with the function of various structures such as fimbriae and virulence pili such as TypeV pilus [53–55]. For the stress response terms, the main family involved was the universal stress protein family. We noticed that more stress response genes were expressed in the wastewater sample. The key environmental factor influencing the dynamics of the microbial consortia and the eventual expression of the universal stress protein in both metagenomes is toxicity [27, 39]. This indicates that there are relative high toxic substances (terephthalic, phthalic, benzoic, trimellitic and acetic acids) and other stressors in the wastewater [50] compared to the activated sludge. More protein biosynthesis is taking place in the activated sludge, indicating that the community is recovering from the high toxic assault in the wastewater that denatured many protein units [32, 43].

Also in the activated sludge the following SEED functions were annotated: protein biosynthesis and clustering-based subsystems (NusA-TFII cluster). The main subsystem involved in the clustering-based subsystems term is the NusA-TFII cluster, whose role is in transcription termination protein NusA. This indicates that more transcription process is taking place in the activated sludge to circumvent the depleted protein load from the high toxic wastewater metagenome [32, 43]. In the wastewater sample we identified the nucleosides and nucleotides function while in both metagenomes the following functions were annotated (1) transportation of manganese, (2) respiratory (soluble cytochrome), (3) cell division, and (4) cell cycle. The Nucleosides and Nucleotides term was annotated only in the wastewater metagenome. The main process is the de novo purine biosynthesis. The de novo purine biosynthetic pathway produces purines which represent the building blocks for DNA and RNA synthesis, provide energy in chemical and redox reactions, and act as signaling molecules in regulatory pathways [56]. The de novo purine pathway consists of ten stepwise reactions that serve to convert phosphoribosyl pyrophosphate to inosine monophosphate. In general, prokaryotes tend to use freestanding single-functional enzymes for the chemical transformation. The main enzyme involve here is Phosphoribosylamine glycine ligase (EC 6.3.4.13) which catalyzes the ligation of glycine and 5-phosphoribosylamine to generate 5-phosphoribosyl glycineamide [57]. This indicates initiation of biosynthesis for primary enzymes needed to commence the chemical transformation of toxics in the wastewater. 

The main membrane transport is in the transportation of manganese. Manganese has a central function in the regulation of stress responses, physiology, and metabolism in prokaryotes [58] and also to be pivotal in pathogenesis [59]. There were no significant differences in the number of reads assigned to both metagenomes. This indicates the need for continuous regulation of stress responses, physiology, and metabolism of the prokaryotes in both metagenomes through manganese transportation. The respiratory term involves soluble cytochrome and functional-related electron carriers. One of the steps of a common pathway for biological energy conversion involves electron transfer between cytochromes [60]. The cytochromes are ubiquitous electron carriers with essential functions in cellular energy and signal transduction. These electron carriers participate in both respiratory and photosynthetic electron-transfer chains [61]. There were more respiratory reads annotated to the wastewater than the activated sludge. This is because the oxidation of terephthalate to acetate and hydrogen is an endergonic reaction unless coupled to methanogenesis reactions that further conversion of the intermediates will result to the final gaseous products [13]. This suggests that more energy is needed for degrading the high toxic concentration in the wastewater sample.

The main cell division and cell cycle was in both communities were the control of cell elongation division cycle, and the particular enzyme involved is the endonuclease III (EC 4.2.99.18). Endonuclease III (EC 4.2.99.18) is a DNA repair enzyme which removes a number of damaged pyrimidines from DNA via its glycosylase activity and also cleaves the phosphodiester backbone at apurinic/apyrimidinic sites via a beta-elimination mechanism [62] with the native containing a single [Fe_4_S_4_]^2+^ cluster. There was no striking difference in the number of reads assigned to each metagenome, although the activated sludge community shows a slight higher annotation of reads. In proliferating cells, DNA damage is detected by sensors that elicit a cellular response which can arrest the cell cycle and repair the damage [63, 64]. This suggests that more prokaryotic cellular proliferation and DNA repairs are taking place to circumvent the depleted microbial loads and the DNA assaults that resulted from the wastewater metagenome.

**Figure 7:**
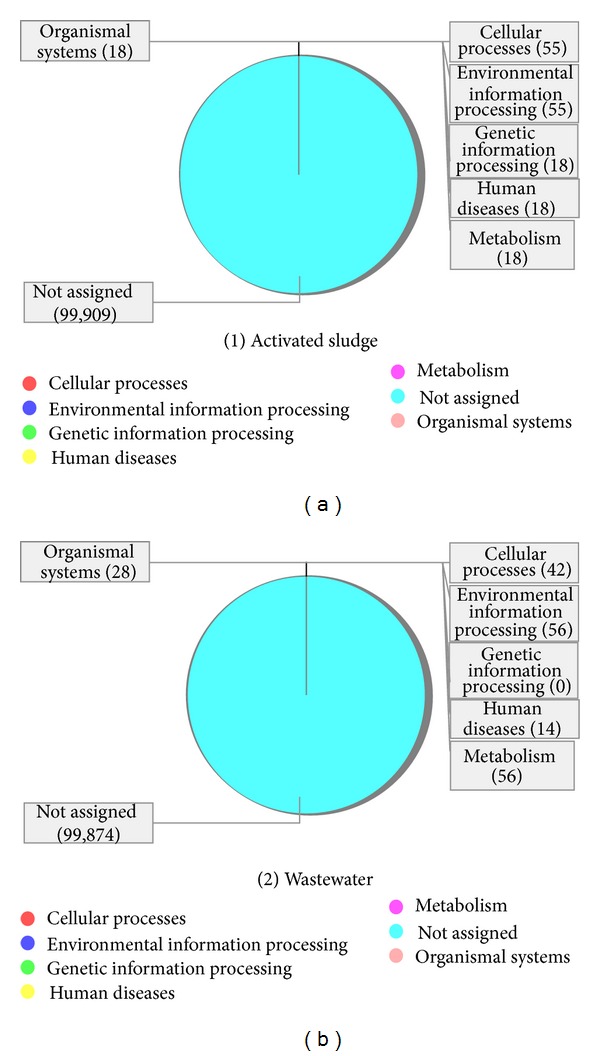
The comparative visualization showing mapping KEGG terms.

**Figure 8:**
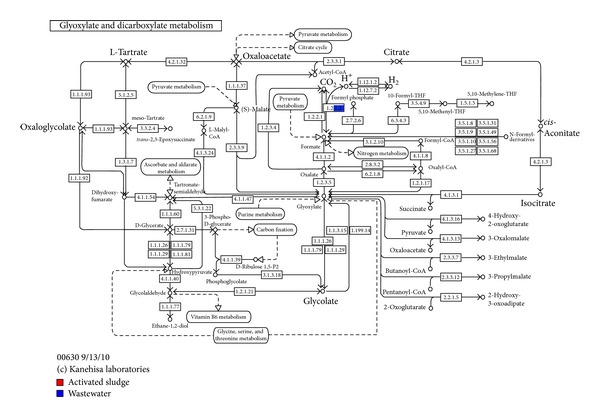
The glyoxylate and dicarboxylate metabolism pathway. The formate dehydrogenase gene (EC: 1.2.1.2) with 28 reads was identified in the wastewater sample but not detected in the activated sludge metagenome. Methanogenic archaea uses formate dehydrogenase gene in methane metabolism pathway to mineralize the intermediate mixture of formate, hydrogen, and acetate to methane and carbon dioxide.

Also the comparative visualization shows the mapping KEGG terms (Figure 7). In this work we shall focus on the four metabolic pathways identified. These pathways include (1) carbohydrate metabolism (glyoxylate and dicarboxylate metabolism); (2) Energy metabolism (methane metabolism); (3) lipid metabolism (glycerophospholipid metabolism), and (4) nucleotide metabolism (purine metabolism). In the glyoxylate and dicarboxylate metabolism pathways, the formate dehydrogenase gene (EC: 1.2.1.2) with 28 reads was identified in the wastewater sample but was not detected in the activated sludge metagenome (Figure 8).

The dehydrogenase gene from most aerobic organisms is devoid of redox-active centers [65] and together with the hydrogen dehydrogenase gene (EC: 1.12.1.2) forms a system previously known as formate hydrogenlyase. Glyoxylate is a toxic intermediate which in humans undergoes oxalate formation [66, 67] with severe consequences for the tissues involved. The glyoxylate cycle is thought to be present in bacteria, protists, plants, fungi, and nematodes but not in other Metazoa [68]. Glyoxylate cycle is a distinct, anaplerotic variant of the tricarboxylic acid (TCA) cycle whose net effect is the conversion of two molecules of acetyl-CoA to succinate gluconeogenesis [68]. Glyoxylate cycle allows the synthesis of macromolecules from dicarboxylates compounds such as ethanol and acetate whose intoxication produces multisystem organ injury [69]. It has been suggested that within the methanogenic consortium, terephthalate is degraded via decarboxylation to an intermediate benzoyl-CoA and later to acetate and hydrogen which are mineralized to methane and carbon dioxide [14, 18]. The presence of this pathway indicates that glyoxylate could be one of the terephthalate intermediate metabolite and together with terephthalate is biochemically transformed in the wastewater sample.

**Figure 9:**
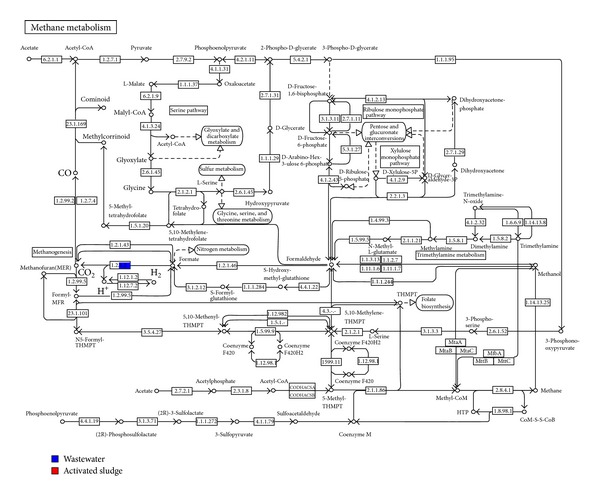
Methane metabolism pathway identified in the wastewater.

In the methane metabolism pathway, the formate dehydrogenase gene (EC: 1.2.1.2) with 28 reads was also identified in the wastewater sample but was not detected in the activated sludge metagenome (Figure 9). In methane metabolism pathway the formate dehydrogenase gene will mineralize the intermediate mixture of formate, hydrogen, and acetate to methane and carbon dioxide by methanogenic archaea [18]. This gene was not detected in the activated sludge sample, which is likely due to the low abundance of reads assigned to Euryarchaeota and methanogenic archaea. Another reason for lack of dehydrogenase gene might be due to low gene coverage encoded by these taxa or due to the absence of terephthalate and toxic intermediate metabolite to be degraded via decarboxylation pathway to methane in the activated sludge metagenome [14, 18].

**Figure 10:**
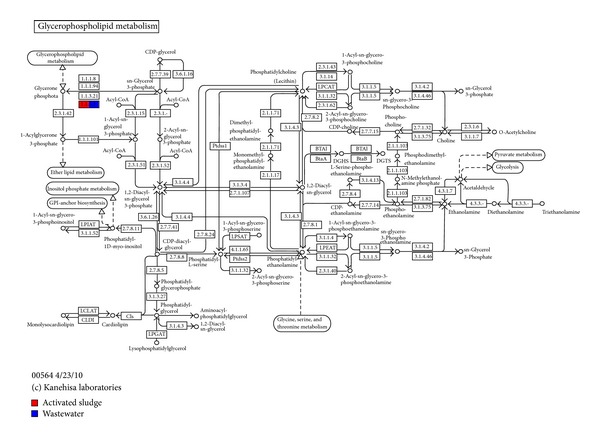
The glycerol 3-phosphate common to both metagenome.

In the glycerophospholipid metabolism pathway, the glycerol 3-phosphate dehydrogenase gene (EC: 1.1.5.3) with 14 reads was identified in the wastewater sample, and also 18 reads were detected in the activated sludge metagenome (Figure 10). The glycerol 3-phosphate dehydrogenase (G3PDH) gene is an oxidoreductase and a key enzyme in the pathway of glycerol synthesis, which converts dihydroxyacetone phosphate (DHAP) to glycerol-3-phosphate [70]. It is a flavin-dependent dehydrogenase and an essential membrane enzyme, functioning at the central junction of glycolysis, respiration, and phospholipid biosynthesis. In bacteria, the enzyme is localized to the cytoplasmic membrane [71]. Glycerol is a small and simple molecule produced in the breakdown of glucose, proteins, pyruvate, triacylglycerols, and other glycerolipids, as well as release from dietary fats. It has long been known to play fundamental roles in several vital physiological processes, in prokaryotes, and eukaryotes and is an important intermediate of energy metabolism [72]. It is the primary energy source for heterotrophic haloarchaea and a major component of “salty” biodiesel waste [73]. Glycerol-3-phosphate dehydrogenase (Gpd1p) is a cytosolic NAD(+)-dependent glycerol 3-phosphate dehydrogenase gene, that is, localizes to peroxisomes and plays a critical role in the cellular response to osmotic stress and a role in redox balance [74].

**Figure 11:**
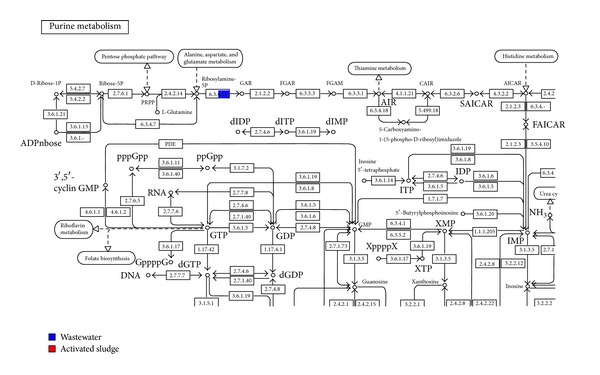
De novo purine biosynthesis pathway identified in the wastewater.

The comparative microbial visualization from our unpublished work indicates that there are generally more reads assigned to the wastewater than activated sludge sample based on the salinity attribute. The high annotation of glycerol-3-phosphate dehydrogenase gene in activated sludge (18 reads) compared to wastewater (14 reads) indicates that the activated sludge community is very sensitive to salinity fluctuations. This correlates with their overexpression of glycerol-3-phosphate dehydrogenase. The glycerol-3-phosphate dehydrogenase (G3PDH) is efficient in protecting against the effect of salt, pH, and temperature stresses [75], and overexpression of the GPD1 gene encoding glycerol-3-phosphate dehydrogenase has been shown to confer high salt stress tolerance and osmoadaptation to microbial cell [76, 77]. In the de novo purine biosynthesis pathway, phosphoribosylamine glycine ligase gene (EC 6.3.4.13) with 14 reads was identified in the wastewater sample but was not detected in the activated sludge metagenome (Figure 11). This might be related to the low abundance of reads assigned to the activated sludge metagenome or the absence of biosynthesis of enzyme building blocks. This suggests that the building blocks of chemical transformation enzymes might have been synthesized in the wastewater metagenome with high terephthalate concentration. Another reason for the absence of enzyme building blocks is the comparative reduction of terephthalate and toxic intermediate metabolite to methane via decarboxylation pathway [14, 18] in the activated sludge. The de novo purine biosynthesis pathway had been explained also in the SEED function.

## 4. Conclusions

The metagenomics concept has been used to analyze the dynamics of potent degrading microbes expressing the universal stress proteins from the terephthalate degrading microbial community. These highly dynamic microbial species that appeared only in the activated sludge of less toxic terepthalate and its derivatives could serve as taxonomic biomarkers for toxic thresholds related to terepthalate and its derivatives. Dynamics of microbial consortium of this nature can have future in biotechnological application in bioremediation such as toxicity monitoring and biosystem augmentation.
